# Feasibility study examining the short-term effects of Sonic Augmentation Technology™

**DOI:** 10.3389/fpsyt.2026.1772405

**Published:** 2026-06-08

**Authors:** Lourdes P. Dale, Audrey N. Dana, Carrie E. Lee, Hannah Lamont, Donnalea Van Vleet Goelz, Caitlin V. Dale, Parmida Nazarloo, Mark McIntosh, Steven P. Cuffe

**Affiliations:** 1 Department of Psychiatry, College of Medicine-Jacksonville, University of Florida, Jacksonville, FL, United States; 2 Department of Neuroscience, College of Medicine, University of Florida, Gainesville, FL, United States; 3 Neuroendocrine Unit, Massachusetts General Hospital, and Department of Medicine, Harvard Medical School, Boston, MA, United States; 4 Southern Methodist University, Dallas, TX, United States; 5 Traumatic Stress Research Consortium, Kinsey Institute, Indiana University, Bloomington, IN, United States; 6 Department of Emergency Medicine, College of Medicine-Jacksonville, University of Florida, Jacksonville, FL, United States

**Keywords:** music, polyvagal theory, oxytocin, biobehavioral state, anxiety, depression, autonomic reactivity

## Abstract

**Background:**

The current study evaluated Sonic Augmentation Technology™ (SAT), a novel, Polyvagal-informed probe that incorporates continuously varying and acoustic features modeled on patterns of autonomic regulation into musical soundscapes. Specifically, the study examined whether SAT led to improvements in self-reported biobehavioral state (e.g. relaxation, breathing slowly, interoceptive clarity), and whether individuals with poorer baseline functioning (i.e., increased autonomic reactivity and anxiety/depression symptoms) reported greater improvements in biobehavioral state following SAT. It also sought to determine whether there would be changes in endogenous oxytocin level, and whether individuals who exhibit larger gains in biobehavioral state would show greater increases in oxytocin.

**Methods:**

The current study examined data obtained from four samples, with 72 participants providing data virtually and 41 participants providing data in-person. Participants completed self-report measures of biobehavioral state, autonomic reactivity, and psychiatric symptomatology (i.e. anxiety and depression). Salivary oxytocin (pg/mL) was assessed via enzyme immunoassay in the participants who completed the study in person, with samples collected pre-and post-SAT.

**Results:**

Following SAT, participants reported significant improvements in total biobehavioral state and the low and high arousal subscales. We also found that participants scoring above the cutoff for autonomic reactivity, anxiety, and depression reported greater increases in total biobehavioral state and decreases in the high arousal subscale. Greater decreases in the low arousal subscale were observed in the participants with increased anxiety. The subset of participants with salivary oxytocin samples exhibited increases in salivary oxytocin.

**Conclusions:**

Thus, our results support the potential of SAT as a low-cost, non-invasive auditory probe for improving wellbeing, especially for clinical populations experiencing autonomic dysregulation and psychiatric difficulties, such as anxiety and depression.

**Clinical trial registration:**

## 1 Introduction

Across cultures and centuries, structured sounds, often in the form of ritualized vocalizations, drumming, chanting, and other acoustic elements, have been interwoven with music in practices of healing, mourning, celebration, and transformation. These ritual sounds did not exist separately from music but complemented and extended it, creating immersive acoustic environments. It is widely believed that early societies engaged with both music and these structured sounds not merely for entertainment, but also as integral tools for enhancing wellbeing (1). Fast-forwarding to the present day, advances in neuroimaging and electrophysiology now lend credence to those early intuitions (2, 3). Accumulating evidence suggests that music, together with structured, ritualized sounds, can exert transformative effects on the brain and body by promoting neuroplasticity, regulating autonomic states, and modulating neural circuits implicated in emotion, cognition, and physical function (3). Accordingly, there is increasing interest in the deliberate use of music and structured sound within therapeutic interventions, with benefits observed whether individuals actively engage in sound making or passively listen to it (3–5).

Sound-based interventions are commonly categorized by this mode of engagement. Active approaches require participation in music-making, whereas passive approaches involve listening alone (4, 6). Both forms appear biologically potent. Active interventions have been associated with significant increases in oxytocin (7) and robust activation of auditory–motor neural sequences (8), while passive interventions may increase oxytocin and reduce cortisol, indicating concurrent effects on affiliative neurobiology and stress physiology (9–11).

Within passive approaches, emerging methods highlight that specific acoustic structures can shape autonomic and emotional outcomes. Vibroacoustic stimulation is an intervention that combines tactile and auditory inputs and has been shown to reduce psychological and physiological stress markers (12). Filtered vocal music, such as that in the Safe and Sound Protocol, has a partial effect on social receptivity and has shown initial data of reduced hypersensitivity to sensory stimuli in adults with autism spectrum disorder (13, 14), and theta-frequency auditory stimulation can promote parasympathetic activation alongside sympathetic withdrawal (15). Yet, many passive “healing frequency” protocols (e.g., 528 Hz) employ static tones rather than modulating acoustic features in ways that track or support homeostatic autonomic regulation (9). To maximize the therapeutic reach of passive sound-based interventions, there is a need to design protocols that may directly engage the autonomic nervous system.

Polyvagal Theory (PVT) provides a neurophysiological framework for understanding how sound-based interventions may foster autonomic regulation. Unlike models centered on cognitive appraisal, PVT hypothesizes that autonomic state regulation is mediated through brainstem systems that integrate visceral afferent input across cardiovascular, respiratory, and gastrointestinal domains. These systems support dynamic coordination of physiological and biobehavioral states. PVT emphasizes neuroception, a reflexive neural process through which the body evaluates environmental and internal cues of risk or safety outside of conscious awareness (16). According to PVT, when the autonomic nervous system detects cues of safety, vagal mechanisms act to inhibit defensive physiology and support social engagement, affect regulation, and autonomic balance (16). It is hypothesized that trauma or chronic stress can retune neuroception toward danger detection even in objectively safe contexts, thus biasing the organism toward survival states (16). Over time, chronic generalized threat reactivity and corresponding autonomic mobilization (e.g., defensive fight/flight reactions) may contribute to reduced behavioral flexibility (17). In addition, persistent maladaptive stress and its associated physiological patterns may contribute to adverse health outcomes, including cardiovascular disease and metabolic syndrome (17).

Oxytocin is a neuropeptide that can modulate threat-related physiology, potentially increasing parasympathetic tone through its influence on cardiac vagal circuits (18). Human studies indicate that oxytocin can enhance heart rate variability, promote vagal recovery, and dampen stress reactivity (19–21). Complementary animal work suggests that lower endogenous oxytocin levels may be associated with decreased prosocial behavior (22). Together, oxytocinergic signaling and autonomic regulation appear to form coordinated, adaptive mechanisms for regulating stress and social approach (19–22). Consistent with this view, reduced oxytocin availability (23) and elevated autonomic reactivity (24–27) have each been linked to greater psychiatric symptom burden across multiple conditions (28). Notably, several behavioral effects attributed to oxytocin (e.g., enhanced social engagement, reduced threat reactivity, and improved affect regulation) overlap with effects reported following sound-based interventions, including listening to music (3). This convergence is plausible given that some neural networks engaged by pleasurable acoustic stimuli also participate in oxytocin processing (7).

One passive, PVT-informed auditory probe is Sonic Augmentation Technology™ (SAT). SAT is based on the premise that affective and visceral difficulties often reflect atypical autonomic regulation across the autonomic nervous system and interrelated systems (e.g., endocrine and immune) supporting homeostasis (29). Within this framework, the temporal structure of physiological processes (e.g., heart rate variability, respiration, gut motility) indexes regulatory capacity, whereas reduced variability is often associated with compromised function (30). This perspective is consistent with network physiology, which conceptualizes physiological systems as dynamically interacting networks whose coupling shifts across states such as rest, stress, and disease (31, 32). Accordingly, adaptive function reflects flexible coordination across systems rather than the activity of any single subsystem.

SAT is not intended to directly stimulate autonomic pathways or replicate canonical cues of safety (e.g., prosodic vocal signals). Rather, SAT provides temporally structured acoustic input hypothesized to support a calm physiological state and facilitate endogenous oxytocin release and autonomic regulation. Supporting these states is important given that prior research suggests that regulation of the oxytocinergic and autonomic systems may be linked to reduced psychiatric symptomatology (19–27). Therefore, SAT may lead to improvements in autonomic regulation and mood similar to those observed in other bottom-up therapeutic modalities, such as yoga (33) and the recently developed Somatic Psychoeducational Intervention (34).

The present study does not evaluate the aforementioned hypothesized mechanisms, which are theoretical, state-mediated, and requiring empirical validation. Rather, it evaluates the feasibility of SAT as an auditory probe with four samples. Because many of these individuals are providers in mental health and other healthcare fields, they may face emotional and physiological strain due to the demands of their work, including secondary traumatic stress (35). SAT may be especially relevant for these individuals and others working in high-demand environments where chronic occupational stress may lead to anxiety, depression, burnout, and moral injury (24, 35).

Despite growing evidence that music and structured sound can modulate both neuroendocrine activity and autonomic state, we are unaware of research examining whether a passive 15-minute auditory probe can elicit an oxytocin response and improve autonomically mediated biobehavioral states (e.g., calm, breathing slowly, interoceptive clarity). The present study addresses this gap by determining whether SAT may be associated with subjective improvements in the primary outcome of biobehavioral state and objective increases in the secondary outcome of salivary oxytocin levels in as subset. In addition, we examined the role of baseline measures (autonomic reactivity, anxiety, and depression) as potential predictors of response to the auditory probe.

Consistent with polyvagal theory, biobehavioral state is conceptualized as a state-dependent construct reflecting the integrated subjective experience of autonomic regulation, including bodily regulation, affective state, and perceived capacity for regulation. This was assessed via a newly developed self-report measure called the Biobehavioral State Index, which was designed to capture dynamic, state-dependent changes in emotional and interoceptive experiences that may change over short time intervals, such as the 15 minutes of SAT. Specifically, the measure includes items indexing bodily regulatory experiences (e.g., breathing patterns, muscular relaxation, physical tension/discomfort), affective states (e.g., calmness, anxiety, irritability, feeling overwhelmed), and perceived regulatory capacities (e.g., ability to quiet thoughts, sense of internal stability). Unlike other measures of current state, such as the State-Trait Inventory of Cognitive and Somatic Anxiety (36), the items in the Biobehavioral State Index include experiences deemed to both be positive (e.g., peaceful and relaxed state, ability to quiet thoughts, breathing slowly, muscular relaxation, awareness of bodily rhythms). It is important to note that the BSI has not yet undergone formal validation, and thus its use in the current study is exploratory and hypothesis-generating.

Thus, the objectives of this feasibility study were to determine whether:

1. The Biobehavioral State Index demonstrates internal consistency and is associated with baseline autonomic reactivity and symptoms of anxiety and depression.
2. SAT is associated with improvements in self-reported biobehavioral state and increases in oxytocin levels.
3. Individuals with poorer baseline functioning (i.e., increased autonomic reactivity, and greater anxiety/depression symptoms) show greater improvements in biobehavioral state following SAT.
4. Participants who report improvements in behavioral state also experience greater increases in oxytocin.

## 2 Materials and methods

### 2.1 Study design

This was a pragmatic, multi-center clinical trial, which examined the benefits of listening to SAT. All study related activities were conducted in accordance with the Declaration of Helsinki and University of Florida’s Institutional Review Board, and were registered at ClinicalTrials.gov (Group 1: IRB202401783, NCT06710886; Group 2: IRB202500241, NCT06902506; Group 3: IRB202401217, NCT06580119; and Group 4: IRB202500959, NCT07065227).

Although two groups included randomized control conditions (Group 2: non-augmented condition; Group 4: Mozart condition), these data are not presented in the current manuscript due to problems with the quality of the data. Specifically, technical difficulties (e.g., auditory issues) affected the online stimulus delivery of the non-augmented condition. In addition, random assignment failed to produce comparable subgroups, as the subgroup administered the Mozart condition included significantly less individuals scoring above the cutoff with respect to psychiatric symptomatology and autonomic reactivity. Thus, the present manuscript reports findings from the SAT condition only, based on within-subject pre–post analyses.

### 2.2 Procedures

#### 2.2.1 Recruitment

Participants in Group 1 (*n* = 13) were attendees of a virtual conference workshop session and were recruited during the session by the study team, who were present for the session, via word of mouth. Participants were eligible to participate if they were attending the virtual workshop session, proficient in English, and between 18 and 89 years of age. Participants were presented with the Qualtrics QR code that directed them to the informed consent form followed by the online survey.

Group 2 (*n* = 59) participants were recruited via mass email containing the study advertisement detailing that participating would involve listening to calming music. The email was distributed using the direct email marketing database of the Polyvagal Institute, which is a not-for-profit educationally focused institute (https://www.polyvagalinstitute.org/). The same study advertisement was also posted on the Polyvagal Institute’s event page. The advertisement included a hyperlink to the scheduled study session held over Zoom. Participants were eligible to participate if they were proficient in English and between 18 and 89 years old. On the day of the scheduled study session, participants were presented with the QR code that directed them to the Qualtrics form that first presented informed consent form followed by the online survey.

Participants in Group 3 (*n* = 25) were part of a larger longitudinal study; however, the current manuscript focuses only on their pre- and post-listening data. Participants, who were child protection workers employed at a local community service agency, were recruited via word of mouth by study staff during an informational Zoom meeting facilitated by the agency, and by mass email containing the study flyer, which stated that participants would listen to calming music. Eligibility criteria included being employed as a child protection worker at the agency, proficient in English, and between 18 and 89 years old. Participants attended one in-person intervention session that was held in a conference room at their place of employment. All participants provided their written informed consent prior to participating in the study and received an e-gift card as compensation for their time participating in-person.

**Figure 1:**
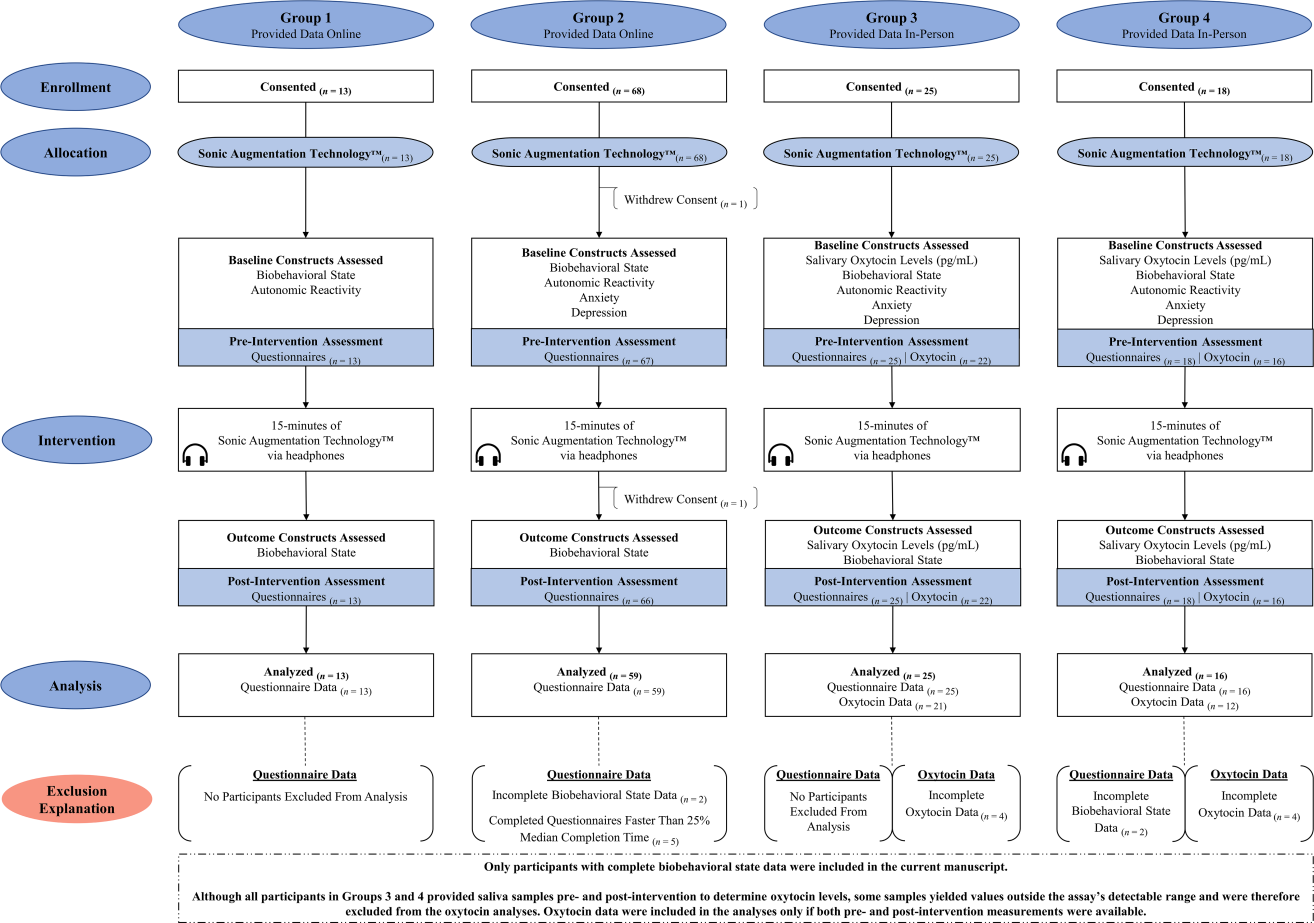
CONSORT Diagram: flow of participants through the study.

Participants in Group 4 (*n* = 16), which consisted of staff from a major medical institution, were recruited through flyers, which advertised that participation would include listening to calming music, distributed via mass email using an employee Wellness listserv and in-person through the hospital’s in-patient and out-patient units. Eligibility criteria included being employed at the medical institution, proficient in English, and aged 18 to 89 years old. Participants attended one in-person intervention session that was held in a conference room at their place of employment. Written informed consent was provided by all participants prior to participating in the study. Participants received an e-gift card as compensation for their time participating in-person. **Figure 1** displays the samples recruited and their flow through the study.

#### 2.2.2 Sonic augmentation technology™

Sonic Augmentation Technology ™ (SAT; Sonocea Inc., USA) is described in U.S. Patent Application No. 18/846,500, titled “System and Method for Regulating Biobehavioral States.” This technology is the intellectual property of Polyvagal Music LLC, a Florida limited liability company, and is utilized under its authorization. The authors of the present study were not involved in the design or development of SAT. Descriptive information regarding SAT was provided by the developers. Consistent with reporting standards for preliminary, proprietary, and device-based research, the manuscript describes the auditory probe in terms of observable stimulus characteristics and study procedures, which are sufficient to support scientific evaluation and replication at the level of implementation, while internal signal-generation methods remain proprietary.

SAT is a 15-minute auditory probe delivered via headphones that is hypothesized to support homeostasis and autonomic biobehavioral state. Given that SAT is a passive auditory experience, it does not require attention or active engagement from participants. SAT differs from conventional music, as it is engineered off grid, without fixed time signatures, repeating measures, or constant tempo, thereby minimizing musical expectation and sustained attentional tracking. SAT provides acoustic patterning hypothesized to reduce sensory vigilance while supporting endogenous regulatory dynamics of the autonomic nervous system. The tempo of the auditory probe varies within a range of approximately 50–80 beats per minute (bpm), while utilizing rapidly changing frequencies varying from 20hz to 5,000hz. It is not constrained to a fixed musical key, scale, or tonal center, rather, it oscillates between the regulated ranges of various endogenous rhythms to promote bodily entrainment of more than one visceral organ or system at a time. The design is influenced by regulated endogenous autonomic regulatory rhythms, including respiration (37), cardiac vagal (38, 39), vasomotor (39), cerebral spinal fluid (39, 40), among numerous other regulated endogenous autonomic rhythms. As an example, SAT purports to cue a regulated heart rate by embedding varying tempos between 60–100 bpm, alongside other modulations, drawing on the concept of bodily entrainment (41). This range aligns with the typical resting heart rate of a healthy human adult, which generally ranges from 60–100 bpm depending on various factors (e.g., activity level and conditioning) (42). This could be presented within SAT as the tempo (i.e., 60–100 bpm) and/or the frequency conversion of that regulated heart rate (i.e., 1-1.7hz). In addition, rhythmic and temporal features are embedded in a non-synchronous manner that reflects the organization and time scales of autonomic regulation without forcing correspondence to any single physiological signal, and without providing paced cues intended to guide or control breathing or other bodily rhythms.

#### 2.2.3 SAT delivery

SAT was prerecorded and played by the interventionist. For Groups 1 and 2, SAT was delivered via audio share on Zoom during virtual group sessions in which participants’ cameras and microphones were turned off/disabled throughout the session. Participants were instructed to wear headphones and to set the volume so that it was not too loud or unpleasant. They were told that the quietest parts of the audio should be just barely audible, as if fading over a hill, while all elements of the audio remained perceptible. Thus, the softer measures were to remain subtle, and louder measures were not to become overpowering. Participation for Groups 3 and 4 occurred in-person within a group setting. The interventionist and playback equipment were positioned behind the participants, and participants were provided with wireless headphones with the volume pre-set by the interventionist to be consistent with the instructions provided to Groups 1 and 2. However, participants were informed that they may adjust the volume to their comfort, provided it remained within the guideline that the softest parts of the audio should be barely audible, the louder parts were not overpowering, and all audio elements remained discernible. Thus, although exact decibels (dB) were not directly measured, instructions and pre-set headphone settings aimed for levels as low as 58 dB, with peaks not intended to exceed a comfortable listening level of approximately 70 dB.

#### 2.2.4 Data collection

Data was collected immediately before (baseline) and after listening to the 15 minutes of the Sonic Augmentation Technology (SAT) auditory probe. At baseline, all participants reported their demographic characteristics (i.e., gender, race, and age) and completed measures assessing their autonomic reactivity and biobehavioral state. Groups 2, 3, and 4 also completed measures examining depression and anxiety symptomatology prior to experiencing the auditory probe. All groups completed the measure assessing biobehavioral state after listening to the music.

Following completion of the pre-assessment survey, participants were presented with a stop sign instructing them not to proceed until they had listened to the full 15 minutes of calming music and were verbally reminded of this instruction. Five participants completed the survey faster than 25% of the median completion time (i.e., in less than 18 minutes) suggesting that they did not comply with the instruction to listen to the music prior to continuing to the post-music assessment. Thus, these responses were excluded from analysis to ensure that the data accurately reflect the intended experimental procedure. Additionally, because the primary focus of the study is exploring differences in biobehavioral state, only participants with complete data on the Biobehavioral State Index were included in the current manuscript. Thus, four participants were excluded from analysis, as they had incomplete data on this measure.

Because Groups 3 and 4 participated in person, all participants in these groups were able to provide salivary samples via a passive drool method before and after listening to SAT to assess oxytocin levels (pg/mL). However, eight samples from eight separate participants yielded values outside the assay’s range of the curve fit and/or the range of the standards (see section 2.5.2 for details), and were therefore excluded from the oxytocin analyses. Oxytocin data were included only when both pre- and post-intervention measurements were available. Consequently, eight participants who provided salivary samples were not included in the oxytocin analysis.

### 2.3 Constructs and measures

#### 2.3.1 Self-report measures

Biobehavioral state was assessed using the Biobehavioral State Index (BSI), an unpublished measure developed by two of our authors (LPD and AND). The version of the BSI administered to all participants, which is displayed in **Appendix A**, asks respondents to indicate via a 7-point Likert scale (0 = *not at all, 3 = somewhat*, to *6 = extremely*) the extent they are currently experiencing the following: *awareness of bodily rhythms*, *breathing slowly*, *relaxation*, *ability to quiet thoughts, peacefulness*, *relaxation*, *anxiety*, *irritability*, *feeling overwhelmed by the demands of life*, *worry about the future*, *feelings of vulnerability*, and *physical pain*.

Although the BSI has not yet undergone formal psychometric validation, analyses were run to determine the dimensions of the measure and the internal consistency of the selected items. As reported in **Supplementary Material File 1**, which details the BSI development history, factor analysis determined that 11 of the 12 items fell into two dimensions (see **Supplementary Table 1**), which we termed low and high arousal subscales. The 11 items, which exclude physical pain, were found to be internally consistent (*α* = 0.82), thus supporting the computation of the total biobehavioral state score. Because the measure includes states that are desirable (e.g., muscular relaxation) and undesirable (e.g., anxiety), the five items detailing undesirable states were reverse coded when summing the items to create the total biobehavioral state. This allowed for higher scores to reflect a more regulated biobehavioral state.

The Body Perception Questionnaire Short Form (43–46) is a self-report measure of autonomic reactivity, which has been validated with sensor-based measures of heart rate variability (46). Participants indicated the frequency of 20 specific bodily sensations suggestive of heightened autonomic reactivity (e.g., *I feel shortness of breath* and *my heart often beats irregularly*) over the past 2-weeks via a 5-point Likert scale (1 = *never* to 5 = *always*). Higher sum scores on this measure reflect greater autonomic reactivity, with a cutoff score of 42 or greater indicating heightened/clinically significant autonomic reactivity. This measure demonstrated strong internally consistent in the current sample (α =.89).

Psychiatric symptomatology was assessed via the 7-item GAD-7 (47) which measures anxiety symptoms, and the 8-item PHQ-8 (48). Both measures ask respondents to indicate how frequently they have been troubled by their respective symptoms in the past 2 weeks via a 4-point Likert-type scale (0 = not at all to 3 = nearly every day). Higher sum scores on each measure indicate greater anxiety and depression symptoms, and sum scores of 10 or higher were used to determine which respondents were above clinical cutoff for both anxiety and depression. Both measures were determined to have strong internal consistency in the current sample (GAD-7, *α* = .90 and PHQ-8, *α* = .82).

#### 2.3.2 Endogenous salivary oxytocin

Participants were instructed to refrain from eating, drinking, smoking, or chewing gum for 30–60 minutes before their session, and to avoid brushing or flossing beforehand to reduce the risk of oral microbleeds, which can contaminate peptide measurements (49). Saliva was collected using a passive drool procedure, which is has previously been used for peripheral oxytocin assessment (49). Participants allowed saliva to pool naturally in the mouth and then expelled it into a Salimetrics Saliva Collection Aid (Item No. 5016.04) fitted to a sterile 2 mL cryovial (Item No. 5004.01), both certified for low temperature biobanking and hormone assays. Immediately following collection, samples were placed on ice and transferred to a −20 °C freezer within one hour, consistent with protocols shown to preserve peptide stability (50). Prior to assay, samples were thawed once and centrifuged at 2500 × g for 20 minutes at 4 °C. Samples were visually inspected following centrifugation, and no samples demonstrated evidence of blood contamination or other quality concerns requiring exclusion. The supernatant was then aliquoted into sterile Eppendorf tubes and returned to a −20 °C freezer until analysis.

Oxytocin concentrations were quantified using a sensitive competitive enzyme immunoassay (ELISA; Arbor Assays, Catalog #K048-H1), which has a lower limit of detection of 16.38 pg/mL and low cross-reactivity with vasopressin and related neuropeptides. Because endogenous salivary oxytocin levels are low, samples were concentrated four-fold to ensure placement on the reliable portion of the standard curve (50). Consistent with studies demonstrating valid salivary oxytocin measurement without extraction (49, 50), samples were not extracted. All samples were assayed in duplicate. Plates were run by a researcher, blinded to group assignment and purpose of the study. Standard curves met manufacturer criteria (R² ≥.99), and intra-assay variability remained within accepted ranges for immunoassays measuring low-abundance peptides in saliva. Specifically, the intra-assay coefficients of variation (CVs) were < 13.80% and inter-assay CVs were < 7.96%. Mean oxytocin concentrations were calculated from duplicate wells for each sample. As previously stated, a total of eight samples were excluded because oxytocin concentrations fell below the lower limit of assay detection, even after the four-fold concentration step. All remaining samples included in the current manuscript exceeded the assay detection threshold.

### 2.4 Statistical analysis

Data were collected on Qualtrics XM and transferred to IBM SPSS Statistics Version 30.0.0.0 (51) for statistical analysis. Manual inspections were conducted to assess the quality of responses. The study excluded responses that were incomplete or faster than 25% of the median completion time (see section 2.2.4 for explanation). Cronbach alpha analyses were run to assess the internal consistency of self-report measures.

Given the relatively small sample size, especially when focusing on the subset with the complete oxytocin data, we chose to elect an alpha cutoff of.05 and not to apply a correction that may risk a Type II error. To minimize the risk of Type I errors, we followed the recommendation by (52) of focusing on results that were both statistically significant (*p* <.05) and at least a small effect size. We followed the proposed effect sizes guidelines (53) for small, medium, and large effects (Pearson’s r = .10,.30, and.50; eta and partial eta square = .01,.06, and.14; respectively).

Descriptive statistics were run to determine the sample demographics characteristics with regard to age, gender, and race, and ANOVA and chi square analyses compared the participants who had oxytocin data (i.e., Groups 3 and 4) to the participants who did not have oxytocin data (i.e., Groups 1 and 2) with respect to identified age, gender, and race. Additional descriptive statistics were run to determine the sample characteristics with regard to baseline autonomic reactivity, anxiety, depression, total behavioral state, the low and high arousal subscales, and oxytocin levels. Pearson correlation analyses (one-tailed) determined the correlation among the baseline measures of autonomic reactivity, anxiety, and depression, and whether these baseline measures were correlated with baseline total biobehavioral state and the low and high arousal subscale scores. Total scores on the self-report measures of autonomic reactivity, anxiety, and depression were used to group the participants into those that did or did not score above the clinical cutoff for each measure.

Descriptive analysis determined what percentage of participants improved regarding their total biobehavioral state and low and high arousal subscale scores. Repeated measures ANOVAs examined whether: 1) there were significant improvements from pre to post SAT in total biobehavioral state and the low and high arousal subscale scores; 2) the differences remained when considering group status; and 3) the change in total biobehavioral state and the low and high arousal subscale scores varied for the participants who scored below and above the cutoff for autonomic reactivity, anxiety, and depression.

With respect to salivary oxytocin levels, subset (*n* = 33) descriptive statistics determined the spread of oxytocin scores at baseline and post-SAT and the percent of participants who increased oxytocin post-SAT. Repeated measures ANOVA determined if there was a statistically significant change in oxytocin levels following SAT. Lastly, descriptive statistics examined if there were visible differences in the in the change in oxytocin levels between the participants who did and did not improve with respect to their total biobehavioral state and the low and high arousal subscale scores.

Exploratory analyses, presented as **Supplementary Material**, focused on the individual items of the BSI. Specifically, Pearson correlation analyses (one-tailed) examined the association between the individual items and the measures of autonomic reactivity, anxiety, and depression. Additionally, repeated measures ANOVAs examined whether pre-post differences occurred with respect to the individual items.

## 3 Results

### 3.1 Description of participants

The current study combined data from four groups: Group 1 (*n* = 13) attended a substance use conference and provided data during a virtual group meeting; Group 2 (*n* = 59) were individuals interested in polyvagal theory who provided in a virtual group meeting; Group 3 (*n* = 25) were child protection workers who provided data in an in-person group setting; and Group 4 (*n* = 16) were medical and nonmedical staff from a major medical institution who provided data in an in-person group setting. Participants (*N* = 113) primarily identified as Caucasian (70.8%), female (81.4%), and ranged in age from 21 to 81 years (*M* = 49.26, *SD* = 13.81).

Subsequent analyses compared the participants who had oxytocin data (i.e., Groups 3 and 4) to the participants who did not have oxytocin data (i.e., Groups 1 and 2) with respect to identified age, gender, and race. ANOVA results indicated statistically significant age differences, *F*(1, 110) =14.50, *p* <.002, η^2^ = .12) whereby the participants with the oxytocin data were younger (*M* = 42.00, *SD* = 13.72; range 21 to 68) than the participants without the oxytocin data (*M* = 52.29, *SD* = 12.75; range 25 to 81). Analyses focused on the 103 participants who reported their gender indicated that the groups did not differ with respect to identified gender, χ^2^(1, *n* = 103) = 1.39, *p* = .450, η^2^ = .12), as the majority of participants in the oxytocin group (*n* = 23, 95.8%) and no oxytocin group identified as female (*n* = 69, 87.3%). However, the groups also differed with respect to percentage of individuals who identified as white, χ^2^(1, *n* = 113) = 14.48, *p* <.001, η^2^ = .36), as the oxytocin group included less individuals who identified as white (*n* = 15, 45.5%) than the no oxytocin group (*n* = 65, 81.3%).

### 3.2 Baseline functioning

Participants varied with respect to their scores on the measures assessing their autonomic reactivity (range 20-60, *M* = 30.55, *SD* = 8.62), anxiety (range 0-18, *M* = 6.17, *SD* = 4.92), and depression (range 20-60, *M* = 30.55, *SD* = 8.62). Pearson correlation analyses found that autonomic reactivity was significantly positively correlated with both anxiety (*r* = .35, *p* <.001) and depression (*r* = .43, *p* <.001), which were also significantly related to each other (*r* = .74, *p* <.001). Nearly half (*n* = 52, 46.0%) of participants scored above the clinical cutoff on at least one of the measures assessing their autonomic reactivity, anxiety, and/or depression. Given an adequate number of participants scored above the clinical cutoff for each of the measures (autonomic reactivity *n* = 37, 32.7%; anxiety *n* = 21, 18.6%; and depression *n* = 21, 18.6%), group comparisons could be made.

Participants varied with respect to their scores on the measures assessing baseline total biobehavioral state (range 6-60, *M* = 32.87, *SD* = 11.22). Similar variability was observed with regard to the low arousal subscale (range 3-36, *M* = 18.65, *SD* = 7.11) and the high arousal subscale (range 0-30, *M* = 15.78, *SD* = 6.90).

There was a significant negative correlation between the baseline total biobehavioral state score and the scores assessing baseline autonomic reactivity (*r* = -.20, *p* = .030) and symptoms of anxiety (*r* = -.72, *p* <.001) and depression (*r* = -.67, *p* <.001). Similarly, there was a significant positive correlation between the baseline high arousal subscale score and the scores assessing baseline autonomic reactivity (*r* = .21, *p* = .029) and symptoms of anxiety (*r* = .69, *p* <.001) and depression (*r* = .59, *p* <.001).With regard to the low arousal subscale, there was negatively correlation between these baseline scores and the measures of baseline anxiety (*r* = -.47, *p* <.001) and depression (*r* = -.50, *p* <.001), but not baseline autonomic reactivity (*r* = -.12, *p* = .196). In addition, significant correlations were found between the measures of baseline functioning and for many of the individual items on the BSI [see **Supplementary Table 2**].

### 3.3 Pre-post change

**Figure 2:**
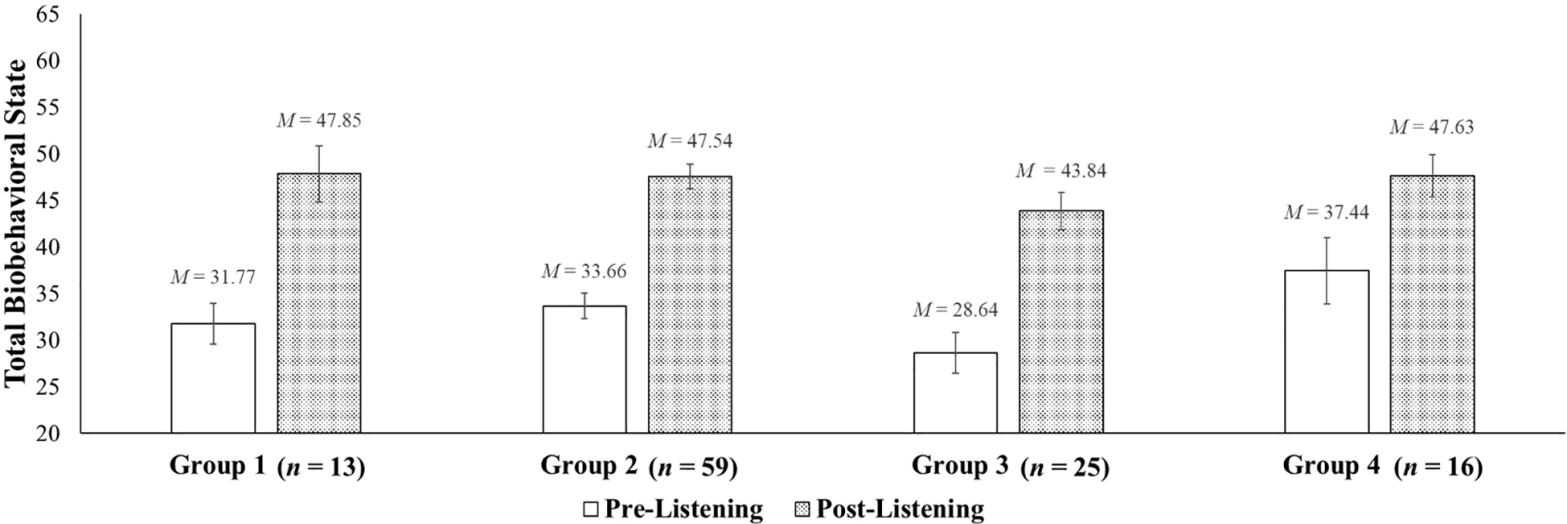
Changes in total biobehavioral state scores. Repeated measures ANOVAs were used to examine changes in total biobehavioral state from pre-listening to post-listening across the four groups, showing consistent improvements with no significant interaction effects. Bar graph displays mean values and error bars represent standard error of mean.

Examination of scores indicated that 88.5% of participants (*n* = 100) reported improvements in total biobehavioral state after SAT. Repeated measures ANOVAs demonstrated significant post-SAT improvements in the total biobehavioral state scores (*F*(1, 112) = 162.02, *p* <.001, ηp^2^ = .59; Pre-Sat *M* = 32.87, *SD* = 11.22 and Post-SAT *M* = 47.63, *SD* = 10.29. As displayed in **Figure 2**, the improvements were consistent for all groups, as there were no significant interaction effects (*F*(3, 109) = 0.23, *p* = .873, ηp^2^ = .01).

Similar results were found both subscales, as 77.0% of participants (*n* = 87) reported improvements on the low arousal subscale and 89.4% of participants (*n* = 101) reported improvements on the high arousal subscale. Repeated measures ANOVAs demonstrated significant post-SAT improvements for the low arousal subscale (*F*(1, 112) = 63.80, *p* <.001, ηp^2^ = .36; Pre-Sat *M* = 18.65, *SD* = 7.11 and Post-SAT *M* = 25.29, *SD* = 6.66) and the high arousal subscale (*F*(1, 112) = 182.31, *p* <.001, ηp^2^ = .62; Pre-Sat *M* = 15.78, *SD* = 6.90 and Post-SAT *M* = 7.67, *SD* = 6.20). In addition, **Supplementary Table 3** details significant post SAT improvements in 10 of the 11 individual items of the BSI and **Supplementary Figure 1** displays mean change scores for each item.

#### 3.3.1 Differences in low and high autonomic reactivity groups

**Figure 3:**
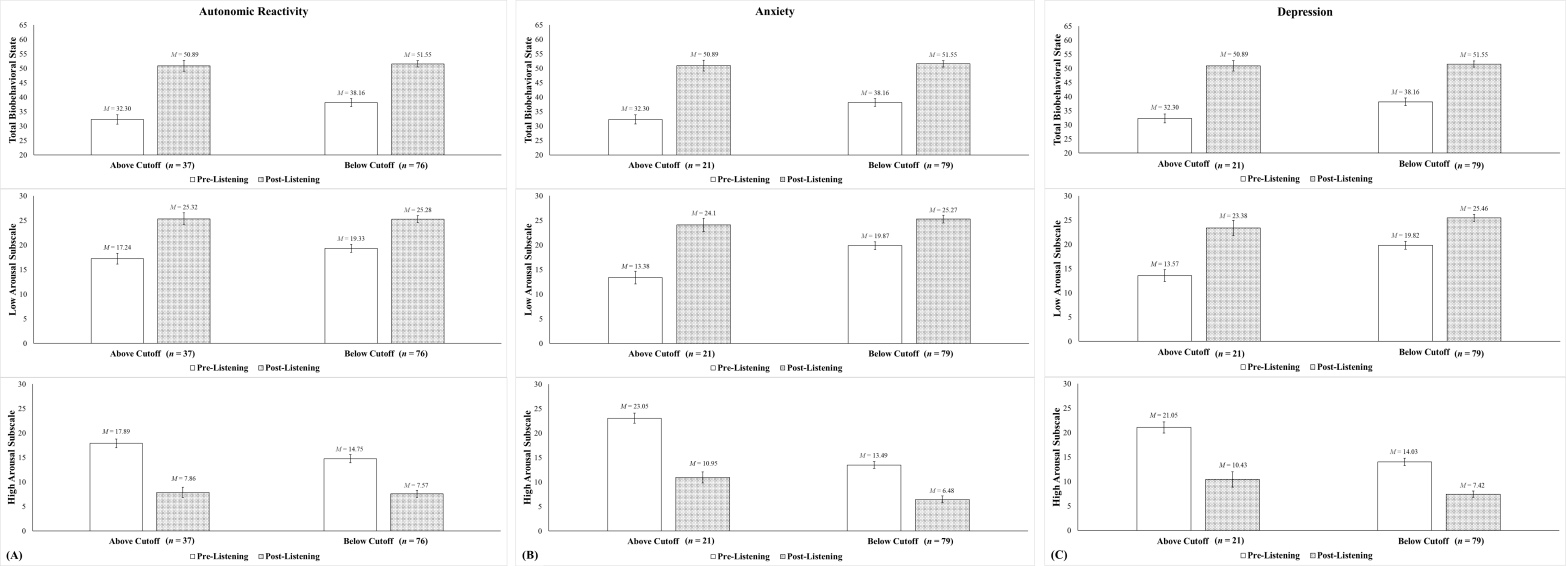
**(A)** Changes in total biobehavioral state score for participants scoring above or below the cutoff for baseline autonomic reactivity. At baseline, 37 participants scored above the cutoff for autonomic reactivity and 76 scored below cutoff. Repeated measures ANOVAs examined whether changes in biobehavioral state total scores and low and arousal subscale scores varied for the participants who scored above and below the cutoff for autonomic reactivity. Participants who scored above cutoff exhibited a greater increase in total biobehavioral state following SAT than the participants who scored below cutoff, with both groups reaching comparable post-SAT levels. Subscale analyses indicated that the statistically significant group differences were driven by the high arousal subscale, as a significant interaction effect was found, whereas no significant interaction effects were found for the low-arousal subscale. Bar graph displays mean values and error bars represent standard error of mean. **(B)** Changes in total biobehavioral state score for participants scoring above or below the clinical cutoff for baseline anxiety. At baseline, 21 participants scored above the cutoff for anxiety and 79 scored below cutoff. Repeated measures ANOVAs examined whether changes in biobehavioral state total scores and low and arousal subscale scores varied for the participants who scored above and below the cutoff for anxiety. Although participants who scored above cutoff exhibited a greater increase in total biobehavioral state following SAT than the participants who scored below cutoff, and they still had statistically significant lower total biobehavioral state scores following SAT. Subscale analyses found that the statistically significant group differences were with respect to both the low and high arousal subscales, as significant interaction effects were found for both subscales. However, the group differences were more pronounced with respect to the high arousal subscale. Bar graph displays mean values and error bars represent standard error of mean. **(C)** Changes in total biobehavioral state score and low and high arousal subscale scores for participants scoring above or below the clinical cutoff for baseline depression. At baseline, 21 participants scored above the cutoff for depression and 79 scored below cutoff. Repeated measures ANOVAs examined whether changes in biobehavioral state total scores and low and arousal subscale scores varied for the participants who scored above and below the cutoff for depression. Although participants who scored above cutoff exhibited a greater increase in total biobehavioral state following SAT than the participants who scored below cutoff, they still had statistically significant lower total biobehavioral state scores following SAT. Subscale analyses indicated that the statistically significant group differences were driven by the high arousal subscale, as a significant interaction effect was found, whereas no significant interaction effects were found for the low-arousal subscale. Bar graph displays mean values and error bars represent standard error of mean.

As reported in **Figure 3a**, repeated measures ANOVAs found significant differences in the pattern of biobehavioral state total scores between participants who scored below and above the cutoff for autonomic reactivity. Significant interaction effects were found with respect to autonomic reactivity, whereby the participants who reported high baseline autonomic reactivity (*n* = 37) exhibited a greater increase in total state scores following SAT as compared to the participants who did not report clinically elevated baseline autonomic reactivity (*n* = 76), (*F*(1, 111) = 4.17, *p* = .044, ηp^2^ = .04). Despite baseline differences, ANOVA results indicated that the participants above the cutoff for autonomic reactivity reached a comparable level post SAT as the group below the cut off, as evident by their mean total biobehavioral scores (*F*(1, 111) = 0.02, *p* = .904, η^2^ = .00). However, as displayed in **Figure 3a**, subscale analyses found that the statistically significant group differences were with respect to the high arousal subscale, as there was a significant interaction effect (*F*(1, 111) = 5.11, *p* = .026, ηp^2^ = .04). In contrast, there was no significant interaction effect with respect to the low arousal subscale (*F*(1, 111) = 1.45, *p* = .230, ηp^2^ = .01).

#### 3.3.2 Differences in low and high anxiety groups

As displayed in **Figure 3b**, significant interaction effects were found with regard to anxiety, as the participants who scored above cutoff with respect to anxiety (*n* = 21) exhibited a greater change in total biobehavioral state scores as compared to the participants who did not report clinically elevated baseline anxiety (*n* = 79; *F*(1, 98) = 12.73, *p* <.001, ηp^2^ = .12). Although the participants scoring above the cutoffs exhibited a greater increase in biobehavioral state after the SAT (as evident by the significant interaction effects), ANOVA results indicated that they still had statistically significant lower total biobehavioral state scores following the SAT (anxiety *F*(1, 98) = 5.23, *p* = .024, η^2^ = .05). As evident in **Figure 3b**. subscale analyses found that the statistically significant group differences were with respect to both the low and high arousal subscales, as significant interaction effects were found (low arousal subscale: *F*(1, 98) = 5.85, *p* = .017, ηp^2^ = .06 and high arousal subscale *F*(1, 98) = 11.92, *p* = <.001, ηp^2^ = .11). However, as evident by the effect sizes, the group differences were more pronounced with respect to the high arousal subscale.

#### 3.3.3 Differences in low and high depression groups

**Figure 3c** displays the significant interaction effects were found with regard to depression, whereby the participants who scored above cutoff with respect to anxiety (*n* = 21) and depression (*n* = 21) exhibited a greater change in total biobehavioral state scores as compared to the participants who did not report clinically elevated baseline depression (*n* = 79; *F*(1, 98) = 6.03, *p* = .016, ηp^2^ = .06). Although the participants scoring above the cutoffs exhibited a greater increase in biobehavioral state after the SAT (as evident by the significant interaction effects), ANOVA results indicated that they still had statistically significant lower total biobehavioral state scores following the SAT (*F*(1, 98) = 5.71, *p* = .019, η^2^ = .06). However, as displayed in **Figure 3c**, the statistically significant group differences were only found with respect to the high arousal subscale, as there was a significant interaction effect (*F*(1, 98) = 4.44, *p* = .038, ηp^2^ = .04). Conversely, there was no significant interaction effect with respect to the low arousal subscale (*F*(1, 98) = 3.52, *p* = .064, ηp^2^ = .04).

### 3.4 Oxytocin Subgroup Analyses

**Figure 4:**
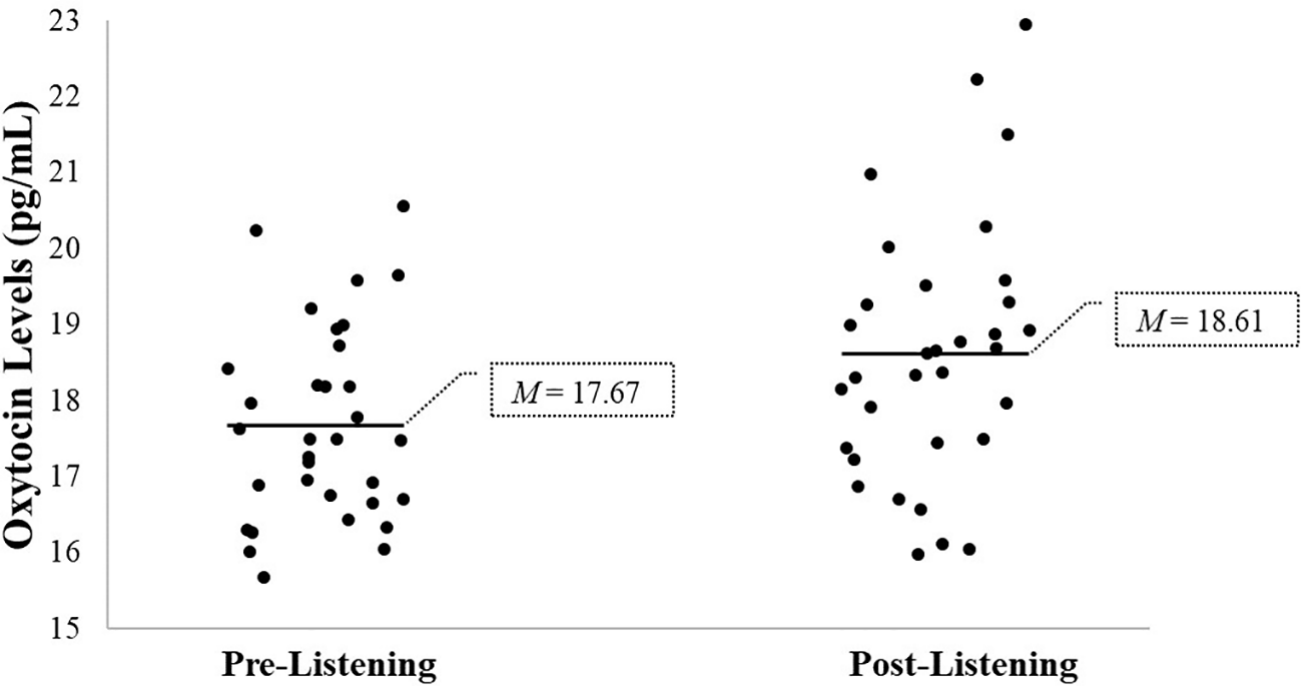
Changes in oxytocin levels. Jitter plot displaying the distribution of oxytocin data pre- and post-listening to SAT for the 33 participants with complete oxytocin data. Participants varied with respect to oxytocin levels pre-listening (range 15.67-20.56, *M* = 17.67, *SD* = 1.28) and post-listening (range 15.98 – 22.95, *M* = 18.61, *SD* = 1.69).

**Figure 4** displays the spread of oxytocin data pre- and post-SAT. Analysis focused on the 33 participants with complete oxytocin data indicated that the participants varied with respect to oxytocin levels pre-SAT (range 15.67-20.56, *M* = 17.67, *SD* = 1.28) and post-SAT (range 15.98-22.95, *M* = 18.61, *SD* = 1.69). Most participants (*n* = 24, 72.7%) experienced an increase in oxytocin levels. Repeated measures ANOVA revealed a statistically significant increase in oxytocin (*F*(1, 32) = 12.99, *p* = .001, ηp^2^ = .29).

Because most participants improved with respect to their scores with respect to total biobehavioral state and the low and high arousal subscales, statistical group comparison could not be run. However, descriptive statistics demonstrated that 22 of 28 participants (78.6%) in the improved total biobehavioral state group exhibited an increase in oxytocin, with the average increase being 1.11 (*SD* = 1.41). In contrast, only 2 of 5 participants (40.0%) in the no increase in total biobehavioral state group exhibited an increase in oxytocin, with the average decrease being -0.05 (*SD* = 1.73).

Similar results were found with respect to the low arousal subscale, as descriptive statistics demonstrated that 21 of 25 participants (84.0%) in the improved total biobehavioral state group exhibited an increase in oxytocin, with the average increase being 1.11 (*SD* = 1.31). In contrast, only 3 of 8 participants (37.5%) in the no increase in total biobehavioral state group exhibited an increase in oxytocin, with the average increase being 0.41 (*SD* = 1.97).

The differences were also observed for the high arousal subscale, as descriptive statistics demonstrated that 23 of 28 participants (82.1%) in the improved total biobehavioral state group exhibited an increase in oxytocin, with the average increase being 1.24 (*SD* = 1.36). In contrast, only 1 of 5 participants (20.0%) in the no increase in total biobehavioral state group exhibited an increase in oxytocin, with the average decrease being -0.78 (*SD* = 0.95).

## 4 Discussion

The present study examined the effects of Sonic Augmentation Technology™ (SAT) on biobehavioral state in a diverse sample of 113 adults drawn from multiple community and professional contexts. The study also examined salivary oxytocin levels in a subset of 33 individuals. The results from this feasibility study offer four primary contributions: 1) they provide initial support for a clinically relevant measure of biobehavioral state (i.e., Biobehavioral State Index) and its corresponding subscales (i.e., low arousal and high arousal subscales), 2) they suggests that targeted passive acoustic stimulation may be associated with improved biobehavioral state and endogenous oxytocin release, 3) they introduces SAT as a potential non-invasive auditory probe for assessing endogenous oxytocin regulation, and 4) they provide initial support for SAT’s potential as an effective auditory probe for clinical populations, as participants with increased levels of baseline anxiety, depression, and autonomic reactivity exhibited greater improvements in biobehavioral state following SAT.

Importantly, this study introduces and provides preliminary validation for the Biobehavioral State Index, which includes a balanced representation of positive and negative valence items, thus reducing directional response bias. Two dimensions were identified via factor analyses we termed the low arousal subscale, which was comprised of the positive valence items, and the high arousal subscale, which was comprised of the negative valence items. This measure demonstrates strong internal consistency and convergent validity and reliable associations with baseline measures of autonomic reactivity, anxiety, and depression symptomatology), which supports its initial construct relevance.

We found that SAT was associated with meaningful improvements in biobehavioral state, which is consistent with research suggesting that passive music interventions may lead to reductions in psychological and physiological stress markers (12) and improvements in emotional regulation (54). Specifically, we found that participants reported statistically significant increases in total biobehavioral state and the low arousal subscale, and decreases in the high arousal subscale. Of note, the improvements were more dramatic for the high arousal subscale, which is suggestive of decreases sympathetic tone, than for the low arousal scale, which is suggestive of atypical parasympathetic regulation.

Consistent with our prior research (30), baseline functioning predicted intervention responsiveness. In the current study, we found that individuals scoring above the cutoff for baseline autonomic reactivity and anxiety/depression symptoms reported more significant increases in total biobehavioral state and decreases in the high arousal subscale (suggestive of decreased sympathetic tone). In contrast, only the individuals scoring above the cutoff for anxiety exhibited significant increases in the low arousal subscale (suggestive of atypical parasympathetic regulation). This suggests that SAT may be especially beneficial for individuals experiencing greater autonomic dysregulation and psychiatric symptomatology. This pattern has direct clinical implications, as baseline profiling may help identify populations in which SAT may be most beneficial.

In as subset, we found that participants demonstrated a statistically significant increase in oxytocin, which has also been observed in prior research (9–11). In addition, we found a trend suggesting a potential association between increases in biobehavioral state and oxytocin, as the majority of participants who reported improvements in total biobehavioral state and the low and high arousal subscales exhibited greater increases in oxytocin than those that did not report these improvements. Although these findings suggest that SAT may serve as an oxytocin provocation and may promote adaptive biobehavioral state shifts, these findings must be interpreted with caution as more research is needed with larger samples.

### 4.1 Limitations and future directions

Several limitations warrant consideration. Given the participants were instructed that they would be listening to calming music, expectancy effects and demand characteristics may have contributed to the observed improvements. Additionally, the study employed a short-term, single-session pre-post design with a brief interval between assessments, which precludes conclusions about the durability of effects. Therefore, future research should refrain from describing SAT as calming and incorporate follow-up assessments to determine whether repeated SAT administrations yield sustained or cumulative benefits. Additionally, the absence of control group limits our ability to draw causal conclusions, and thus future trials should include a control group to isolate the effects of the intervention and allow for causal inferences.

Because the sample was predominantly female, our findings cannot be generalized across sex/gender. This is particularly relevant to the oxytocin findings, as prior research (55) suggests sex differences in oxytocin levels. To address this limitation, future research should examine SAT with a more demographically diverse sample and explore potential sex/gender differences in intervention response.

Although our study collected useful information about baseline functioning, it may have been useful to inquire about their musical listening habits and genre preferences to determine whether these impact the intervention response. It may also have been helpful to ask about their perception of how they felt during the 15-minute SAT exposure. Although the BSI was found to be a reliable measure in the current sample that was sensitive to short-term pre-post changes, findings based on this measure are provisional. Future research is needed to establish factor structure, test-retest reliability, convergent/divergent validity, and sensitivity to change across contexts. Moreover, future studies should assess the effects of SAT using established subjective measures, such as the State-Trait Inventory for Cognitive and Somatic Anxiety (33), and objective measures of autonomic functioning, such as heart rate variability.

Although SAT was associated with significant increases in oxytocin for those with complete oxytocin data, replication in a larger sample is needed to confirm the reliability and magnitude of this effect. Additional data will increase statistical power to evaluate whether oxytocin changes covary with improvements across specific biobehavioral domains and to better characterize individual differences in neuroendocrine responsiveness.

## 5 Conclusions

This study advances the field by demonstrating that SAT, a polyvagal informed auditory probe, is associated with significant improvements in self-perceived biobehavioral state via the Biobehavioral State Index. Individuals with heightened baseline physiological distress (i.e., scoring above the cutoff for autonomic reactivity, anxiety, and depression) exhibited the greatest gains, suggesting that SAT may be especially effective for emotionally challenged individuals. Although preliminary, participants exposed to SAT exhibited increases in endogenous oxytocin. Given these results are based on a small subset, more research is necessary to determine whether SAT is an effective auditory probe that for stimulating the endogenous oxytocin system. Thus, SAT may offer a low-cost, scalable, and biologically grounded method for enhancing psychological well-being. The convergence of improved biobehavioral state and oxytocin release provides a coherent target for future efficacy and mechanistic studies of SAT.

## Data Availability

The datasets presented in this article are not readily available because individuals could potentially be identified through demographic characteristics in our small subsamples. Requests to access the datasets should be directed to Lourdes.Dale@jax.ufl.edu.

## Appendix A

**Biobehavioral State Index**

Dale, L. D. & Dana, A. N. (2025).

Please review the list below and indicate to what extent you currently experience the following.

**Table:**

0 Not at all	1	2	3 Somewhat	4	5	6 Extremely

1. Ability to quiet thoughts

2. Awareness of rhythms in the body

3. Breathing slowly

4. Muscular relaxation

5. Physical pain*

6. Peaceful

7. Relaxed

8. Anxious*

9. Irritable*

10. Overwhelmed by the demands of life*

11. Worried about the future*

12 Feeling vulnerable*

**Scoring:**

Items may be evaluated individually (specific biobehavioral states) or combined into a total score representing total biobehavioral state. The total score is calculated by summing all items.

Items marked with an asterisk (*) are reverse-scored when calculating the total score. These items can be examined using their raw scores at the individual level but must be reverse-scored before being included in the total score.

**Total score range**: 0–72.

**How to cite**: Dale, L. D. & Dana, A. N. (2025). *Biobehavioral State Index*.
