# Supplementary material for: Feasibility study examining the short-term effects of Sonic Augmentation Technology™
Source: Front Psychiatry. 2026 Jun 8;17:1772405. doi: 10.3389/fpsyt.2026.1772405 (PMC13284157; doi:10.3389/fpsyt.2026.1772405)
Supplement: Supplementary file 1 [file Supplementaryfile1.docx]

**Supplementary Material**

**File 1**

**Development of the Biobehavioral State Index**

The original version of the Biobehavioral State Index was called the termed the Benefits List in Institutional Review Board and Clinical Trial documents. It included 20 items that were believed to be benefits from hearing calming music. Specifically, some of the items were derived from a measure that focused on 12 potential benefits of yoga called the Yoga Experiences Scale [1]. Other items were derived from preliminary reports from individuals who listened to SAT as part of conference workshops, such as the 35^th^ Annual Boston International Trauma Conference, which occurred in 2024 prior to study conceptualization. Together, a list of 20 items was compiled to create Version 1 of the Benefits Scale.

Preliminary data on the 20 items were assessed to determine internal consistency. Based on Cronbach alpha analysis, the number of items was reduced to the 12 internally consistent items that were included in Version 2 [see Appendix A]. Thus, Version 1 was administered to groups 1 through 3 and version 2 was administered to group 4.

An exploratory factor analysis using a varimax rotation was conducted on the 12 items in Version 2 administered to all 4 groups to examine the underlying factor structure. Following the suggestions of Comrey and Lee [2], a factor loading cutoff of 0.55 was applied, as values from 0.55 to 0.63 are considered “good,” and thus variables with loadings below this threshold were removed. The Kaiser-Meyer-Olkin index was .79 and Bartlett’s test of sphericity was significant, χ²(66) = 590.07, *p* < .001, supporting the adequacy of the data for factor analysis. As evident in the varimax rotated factor loadings presented in Table 1, one item (i.e., physical pain) that failed to load significantly on either factor was removed from the analysis (factor loading = 0.41). Two factors emerged, which accounted for 56.79% of the total variance. Based on this analysis, only the 11 items with good factor loading scores were included in the current manuscript.

We created two subscales based on the dimensions identified through factor analysis. The first dimension included items believed to reflect a low arousal or increased parasympathetic state (i.e., ability to quiet thoughts, awareness of rhythms in the body, breathing slowly, muscular relaxation, peaceful, and relaxed). The second dimension included items believed to reflect a high arousal or increased sympathetic state (i.e., anxious, irritable, overwhelmed by the demands of life, worried about the future, and feeling vulnerable). These subscales were strongly correlated with the corresponding factor scores derived from the analysis (low arousal subscale: *r* = .98, *p* < .001; high arousal subscale: *r* = .97, *p* < .001), supporting their validity as representations of the underlying constructs.

Total biobehavioral scores were calculated by summing the following items: ability to quiet thoughts, awareness of rhythms in the body, breathing slowly, muscular relaxation, peaceful, relaxed, anxious (reversed), irritable (reversed), overwhelmed by the demands of life (reversed), worried about the future (reversed), feeling vulnerable (reversed).

**References**

[1] Dale, Lourdes P., Laura E. Carroll, Gillian C. Galen, Rachel Schein, Amanda Bliss, Allison M. Mattison, and William P. Neace. 2011. “Yoga Practice May Buffer the Deleterious Effects of Abuse on Women’s Self-Concept and Dysfunctional Coping.” *Journal of Aggression, Maltreatment & Trauma* 20 (1): 90–102. doi:10.1080/10926771.2011.538005.

[2] Comrey, A. L., & Lee, H. B. (2013). A first course in factor analysis. Psychology Press. https://doi.org/10.4324/9781315827506

**Table 1**

*Factor Analysis of Biobehavioral State Index*

|  | **Factor 1** | **Factor 2** |
| --- | --- | --- |
| **Low Arousal Subscale** |  |  |
| Awareness of bodily rhythms​ | .71^E^ |  |
| Breathing slowly​ | .83^E^ |  |
| Muscular relaxation ​ | .77^E^ |  |
| Relaxed​ | .72^E^ |  |
| Peaceful​ | .64^G^ |  |
| Ability to quiet thoughts​ | .58^G^ |  |
|  |  |  |
| **High Arousal Subscale** |  |  |
| Anxious​ |  | .74^E^ |
| Irritable​ |  | .76^E^ |
| Feeling vulnerable​ |  | .68^VG^ |
| Overwhelmed by life’s demands ​ |  | .82^E^ |
| Worried about the future​ |  | .78^E^ |

.55 < x <.63 = good (G), .63 < x < .71 = very good (VG), .71 < x = excellent (E)

**Table 2**

*Baseline Correlation Among the Individual Items of the Biobehavioral State Index and Autonomic Reactivity, Anxiety, and Depression*

|  | **Autonomic Reactivity** | | **Anxiety** | | **Depression** | |
| --- | --- | --- | --- | --- | --- | --- |
|  | ***r*** | ***p*** | ***r*** | ***p*** | ***r*** | ***p*** |
| **Low Arousal Subscale ​** |  |  |  |  |  |  |
| Awareness of bodily rhythms​ | -.01 | .458 | -.10 | .165 | -.21^S^ | .020 |
| Breathing slowly​ | -.01 | .447 | -.21^S^ | .017 | -.24^S^ | .007 |
| Muscular relaxation ​ | -.06 | .283 | -.30^M^ | .001 | -.36^M^ | < .001 |
| Relaxed​ | -.14 | .065 | -.60^L^ | < .001 | -.56^L^ | < .001 |
| Peaceful​ | -.19^S^ | .023 | -.57^L^ | < .001 | -.60^L^ | < .001 |
| Ability to quiet thoughts​ | -.15 | .052 | -.40^M^ | < .001 | -.31^M^ | < .001 |
|  |  |  |  |  |  |  |
| **High Arousal Subscale** |  |  |  |  |  |  |
| Anxious​ | .19^S^ | .021 | .57^L^ | < .001 | .43^M^ | < .001 |
| Irritable​ | .15 | .062 | .53^L^ | < .001 | .40^M^ | < .001 |
| Feeling vulnerable​ | .17 | .039 | .44^M^ | < .001 | .40^M^ | < .001 |
| Overwhelmed by life’s demands ​ | .20^S^ | .017 | .64^L^ | < .001 | .60^L^ | < .001 |
| Worried about the future​ | .10 | .149 | .54^L^ | < .001 | .48^M^ | < .001 |

Small Effect (indicated by superscript S): Pearson’s *r*≥ 0.10, Medium Effect (indicated by superscript M): Pearson’s *r* ≥ 0.30, Large Effect (indicated by superscript L): Pearson’s *r* ≥ 0.50. Only results that are at least a small effect and are statistically significant carry a superscript letter.

**Table 3**

*Change in The Individual Items of the Biobehavioral States Index*

| **​** | **Pre**  ***M*​(*SD*)** | **Post**  ***M*​(*SD*)** | ***F*​** | ***p*** | **η_p_^2^​** |
| --- | --- | --- | --- | --- | --- |
| **Low Arousal Subscale ​** |  |  |  |  |  |
| Awareness of bodily rhythms​ | 3.24 (1.86) | 3.85 (1.64) | 9.27 | .003 | .08^M^ |
| Breathing slowly​ | 3.09 (1.75) | 4.42 (1.29) | 51.76 | < .001 | .32^L^ |
| Muscular relaxation ​ | 2.83 (1.55) | 4.19 (1.45) | 55.95 | < .001 | .33^L^ |
| Relaxed​ | 2.91 (1.55) | 4.53 (1.49) | 89.08 | < .001 | .44^L^ |
| Ability to quiet thoughts​ | 3.26 (1.43) | 4.60 (1.36) | 68.66 | < .001 | .38^L^ |
| Awareness of bodily rhythms​ | 3.32 (1.65) | 3.70 (1.65) | 3.53 | .063 | .03 |
|  |  |  |  |  |  |
| **High Arousal Subscale** |  |  |  |  |  |
| Anxious​ | 3.09 (1.73) | 1.45 (1.70) | 86.51 | < .001 | .44^L^ |
| Irritable​ | 2.74 (1.71) | 1.00 (1.48) | 95.79 | < .001 | .46^L^ |
| Feeling vulnerable​ | 3.04 (1.71) | 2.16 (1.74) | 30.52 | < .001 | .21^L^ |
| Overwhelmed by life’s demands ​ | 3.47 (1.85) | 1.50 (1.65) | 142.06 | < .001 | .56^L^ |
| Worried about the future​ | 3.44 (1.87) | 1.55 (1.62) | 116.17 | < .001 | .51^L^ |

*N* = 113

η_p_^2^ = Partial eta squared. Small Effect (indicated by superscript S): η_p_^2^ ≥ 0.01, Medium Effect (indicated by superscript M): η_p_^2^ ≥ 0.06, Large Effect (indicated by superscript L): η_p_^2^ ≥ 0.14.

Only results that are at least a small effect and are statistically significant carry a superscript letter.

**Figure 1**

*
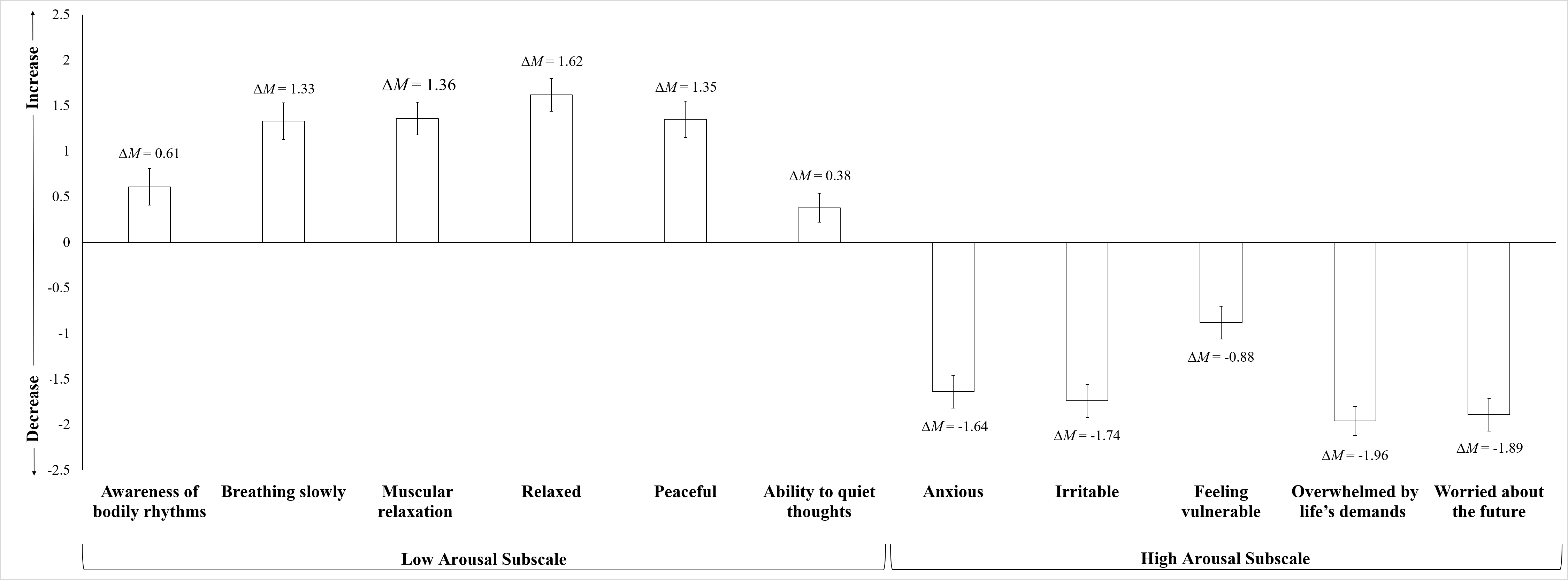
Change in The Individual Items of the Behavioral State Index*

**Legend.** Bar graph displays mean change scores for each item of the Biobehavioral State Index. Error bars represent standard error of mean.
